# Role of EPA in Inflammation: Mechanisms, Effects, and Clinical Relevance

**DOI:** 10.3390/biom12020242

**Published:** 2022-02-01

**Authors:** Rosalia Crupi, Salvatore Cuzzocrea

**Affiliations:** 1 Department of Veterinary Sciences, University of Messina, 98168 Messina, Italy; rcrupi@unime.it; 2 Department of Chemical, Biological, Pharmaceutical and Environmental Sciences, University of Messina, 98166 Messina, Italy

**Keywords:** inflammation, eicosapentaenoic acid, atherosclerosis

## Abstract

Many chronic inflammatory processes are linked with the continuous release of inflammatory mediators and the activation of harmful signal-transduction pathways that are able to facilitate disease progression. In this context atherosclerosis represents the most common pathological substrate of coronary heart disease, and the characterization of the disease as a chronic low-grade inflammatory condition is now validated. The biomarkers of inflammation associated with clinical cardiovascular risk support the theory that targeted anti-inflammatory treatment appears to be a promising strategy in reducing residual cardiovascular risk. Several literature data highlight cardioprotective effects of the long-chain omega-3 polyunsaturated fatty acids (PUFAs), such as eicosapentaenoic acid (EPA). This PUFA lowers plasma triglyceride levels and has potential beneficial effects on atherosclerotic plaques. Preclinical studies reported that EPA reduces both pro-inflammatory cytokines and chemokines levels. Clinical studies in patients with coronary artery disease that receive pharmacological statin therapy suggest that EPA may decrease plaque vulnerability preventing plaque progression. This review aims to provide an overview of the links between inflammation and cardiovascular risk factors, importantly focusing on the role of diet, in particular examining the proposed role of EPA as well as the success or failure of standard pharmacological therapy for cardiovascular diseases.

## 1. Overview of Inflammation

Inflammation is classified as “normal defense mechanism” able to preserve the host from both infections and insults; the involvement of the inflammatory process helps to restore homeostasis at damaged or infected sites. Cardinal signs of inflammation are redness, swelling, heat, pain, and loss of function, and it sees the presence and interaction of different cell types with recruitment of several chemical mediators. If the inflammatory response is well regulated it resolves quickly and without causing damage to the organism, which overall involve the activation of negative feedback mechanisms such as the secretion of anti-inflammatory mediators, the inhibition of pro-inflammatory signaling cascades, shedding of receptors for inflammatory mediators, and activation of regulatory cells [1]; otherwise damage to organs and tissues can occur. Inflammatory mediators normally play a defensive role in the defense of the host; however, if the production of these mediators is not regulated, tissue damage can be caused, inducing the development of different pathologies. Literature data have reported, in fact, that several chronic inflammatory processes are linked with the continuous release of inflammatory mediators and the activation of harmful signal-transduction pathway that facilitate disease progression. In these cases, there are high concentrations of inflammatory markers and cells both at the primary site of damage and at the systemic level, as occurs in cardiovascular disease or rheumatoid arthritis. Recent studies have shown that the condition of low-grade chronic inflammation is decisive in the obesity state, metabolic syndrome, and cardiovascular diseases [2].

**Figure 1:**
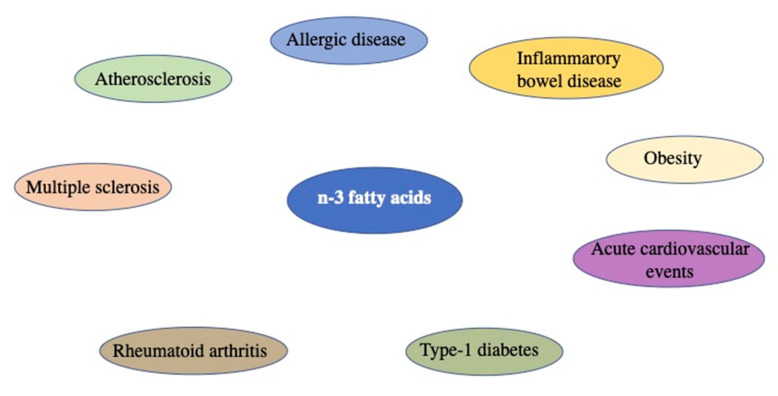
Representative image of inflammatory diseases in which marine n-3 fatty acids might be of benefit.

For the aforementioned concepts, inflammation is classified as the lowest common denominator of numerous pathologies (Figure 1).

Classical events that characterized the inflammatory response include the following:

- An increased blood supply to the site of inflammation.
- Elevated capillary permeability due to retraction of endothelial cells.
- Leukocyte migration from the capillaries into the surrounding tissue. This process is facilitated by the release of chemotactic factors from the site of inflammation and from the upregulation of adhesion molecules on the endothelium.
- Release of mediators from leukocytes at the site of inflammation.

### Lipid Mediators

There are different lipid mediators (e.g., leukotrienes (LTs), prostaglandins (PGs), peptide mediators (e.g., cytokines), amino acid derivatives (e.g., histamine), reactive oxygen species (e.g., superoxide), and enzymes (e.g., matrix proteases) in relationship to the anatomical site involved, cell type involved, nature of the inflammatory stimulus, and the stage during the inflammatory response. Among the mediators involved in the development of inflammatory processes, the first group is represented by toll-like receptors (TLRs), membrane proteins located on macrophages, and dendritic cells. The receptors have the specificity to recognize the molecular patterns that are associated with pathogens (PAMP) and can also identify the endogenous signals that are activated during tissue or cellular damage related to danger (DAMPS). The second group of mediators is represented by the arachidonic acid (AA) mediators. The phosphorylase enzyme is able to act on membrane phospholipids releasing arachidonic acid. The arachidonic acid can metabolize either through the cyclooxygenase pathway or the 5-lipoxygenase pathway. Through the action of cyclooxygenase, the mediators of PGs are formed, which are prostaglandin D2 (PGD2) and thromboxane, which are bronchoconstrictor prostaglandins, and bronchoprotective or inhibitory prostaglandin E2 (PGE2) and prostacyclin. LT is formed from the 5-lipoxygenase pathway [3]. The third group of mediators is represented by mast cells, cells distributed throughout the body.

Mast cells are immune cells of the myeloid lineage and are present in connective tissues throughout the body. The activation and degranulation of mast cells significantly modulates many aspects of physiological and pathological conditions in various settings. With respect to normal physiological functions, mast cells are known to regulate vasodilation, vascular homeostasis, innate and adaptive immune responses, angiogenesis, and venom detoxification. On the other hand, mast cells have also been implicated in the pathophysiology of many diseases, including allergy, asthma, anaphylaxis, gastrointestinal disorders, many types of malignancies, and cardiovascular diseases. The characteristic of these cells is to undergo a degranulation process when activated by tissue damage; pro-inflammatory molecules are released from the granules such as histamine, tumor necrosis factor (TNF), kinin, and leukotrienes [4].

## 2. Inflammation in Cardiovascular Diseases: Focus on Atherosclerosis

All cardiovascular diseases are characterized by the presence of an inflammatory environment often resulting from comorbid pathologies such as diabetes mellitus and arterial hypertension. These are then associated with additional risk factors which are represented by the emotional component and lifestyle. Atherosclerotic cardiovascular disease (ASCVD) is the leading cause of morbidity and mortality globally. In the United States, nearly 808,000 people died from ASCVD in 2014, translating to about 1 in every 3 deaths [5], and the death rate from ASCVD increased by 1% in 2015, the first since 1999 [6]. ASCVD is defined as acute coronary syndrome, stable angina, history of myocardial infarction, coronary or other revascularization, ischemic stroke, transient ischemic attack, or peripheral arterial disease presumably of atherosclerotic origin. Atherosclerosis can also be considered a chronic, immuno-inflammatory, fibroproliferative disease of large and medium-sized arteries fueled by lipids. The study carried out by Anichkov, on rabbits who were fed a diet rich in fatty acids, highlighted the importance and involvement of dyslipidemic phenomena in the development of cardiovascular diseases [7]. Arteriosclerosis is a composite disease in which the inflammatory process is found on the one hand, but on the other, it is also a lipid disorder. Indeed, the Framingham Heart Study in the 1950s demonstrated that hypercholesterolemia accelerates atherosclerosis in humans [3]. In the study of cardiovascular diseases, great importance was given to the thrombotic aspect, which still represents the greatest risk in the management of low-grade systemic inflammation; in fact, there are numerous studies that underline the link between the mediators of inflammation and platelet activation [8].

Formation and rupture of atherosclerotic plaque are closely connected with the development of the inflammatory process. In particular, the lipoprotein stimulates both chemokines and endothelial cells that facilitate the recruitment of lymphocytes and adherent monocytes to the forming lesion site; growth factors and cytokines formed by the inflammatory intima stimulate the differentiation of monocytes into macrophages linked by the upregulation of the TLRs, in particular TLR4 and scavenger receptors; the macrophages activated form foam-like cells or stimulate an inflammatory cascade that lead to atherosclerotic plaque formation [9].

## 3. Fatty Acids: Composition and Role in Inflammatory Processes

Fatty acids are long-chain hydrocarbons and important components of lipids in plants, animals, and microorganisms. Among several role of fatty acids, it was demonstrated that they are able to influence inflammatory processes through several mechanisms by acting, for example, via the cell surface and intracellular receptors/sensors that regulate both gene expression patterns and inflammatory cell signaling. Alterations in fatty acid compositions of cell membranes modulates membrane fluidity, lipid raft formation, and cell signaling leading to altered gene expression. A fatty acid consists of a straight chain of an even number of carbon atoms, with hydrogen atoms along the length of the chain and at one end of the chain and a carboxyl group (―COOH) at the other end. They can be separated into four categories: saturated, mono-unsaturated, polyunsaturated, and *trans* fats. In particular, if the carbon-to-carbon bonds are all single, the acid is saturated; if any of the bonds is double or triple, the acid is unsaturated and is more reactive.

A fatty acid containing two or more double bonds is called a polyunsaturated fatty acid (PUFA). Saturated fatty acids do not contain double bonds in the acyl chain. A few fatty acids have branched chains; others contain ring structures (e.g., prostaglandins). Fatty acids are not found in a free state in nature; commonly, they exist in combination with glycerol in the form of triglycerides. Two principal families of PUFAs are known, namely the n-6 (or omega-6) and the n-3 (or omega-3) (Table 1).

Linoleic acid (18: 2n-6) and α-linolenic acid (18: 3n-3) belong to these families, which have the particularity of not being able to be synthesized by mammals. Vegetable oils contain high amounts of linoleic acid. Green plants and some vegetable oils, on the other hand, contain α-linolenic acid.

Importantly, although linoleic and α-linolenic acids cannot be synthesized by humans, they can still be metabolized into other fatty acids. In particular, linoleic acid can be converted via γ-linolenic acid (18: 3n-6) and dihomo gamma-linoleic acid (20: 3n-6) into arachidonic acid (20: 4n-6); it is also possible to convert α-linolenic acid in eicosapentaenoic acid (20: 5n-3; EPA). The specific chemical structure of EPA reflects important biological characteristics. EPA can modify the physical properties of cellular membranes by substituting the omega-6 fatty acid arachidonic acid (AA; 20:4, n-6) in membrane phospholipids. Moreover, from the metabolism of EPA, antithrombotic and anti-inflammatory lipid mediators can be generated, which are in stark contrast to the prothrombotic and pro-inflammatory factors that are produced by arachidonic acid (AA) [12,13]. EPA can be further metabolized, giving rise to docosapentaenoic acid (22: 5n-3; DPA) and docosahexaenoic acid (22: 6n-3; DHA).

### Fatty Acid Sources

Sources of fatty acids include fruits, vegetable oils, seeds, nuts, animal fats, and fish oils. Essential fatty acids, such as omega-3 fatty acids, serve important cellular functions. They are a necessary part of the human diet because the body has no biochemical pathway to produce these molecules on its own. Particular linoleic and α-linolenic acids are introduced through diet. Arachidonic acid is found in meats, and intake is estimated to be between 50 and 500 mg/day. Fish, especially fatty fish, are rich in EPA, DPA, and DHA. Membrane phospholipids consist of basic units of polyunsaturated fatty acids. Preclinical studies, conducted on laboratory guinea pigs fed according to standard feeding, showed a high content of arachidonic acid (20: 4n-6) and a low content of eicosapentaenoic acid (20: 5n-3; EPA) and docosahexaenoic acid (22: 6n-3; DHA) in the phospholipid composition of tissue lymphocytes [14], peritoneal macrophages [15,16], alveolar macrophages [17], Kupffer cells, and alveolar neutrophils [18]. If the diet of animals was enriched with fish oil, rich in EPA and DHA, there is an accumulation of these in membrane phospholipids both in lymphocytes [14] and in macrophages [19,20], and in Kupffer cells [17]. The even more interesting data that emerges is represented by the decrease in the content of arachidonic acid. Studies conducted on membrane phospholipids of cells such as neutrophils, lymphocytes, and monocytes of patients who consume typical Western diets contain about 10–20% of fatty acids such as arachidonic acid, with about 0.5–1% EPA and about 24% DHA [21,22,23]. The fatty acid composition of these cells can vary by increasing the intake of marine n-3 fatty acids [23,24]. This occurs in a dose–response mode [23] and over a period of days to weeks, with a new steady-state composition achieved within approximately 4 weeks. There are several mechanisms through which fatty acids can influence inflammatory processes, in particular the following:

- By taking fatty acids we can alter the intracellular concentrations of lipoproteins, metabolites, complex lipids, and hormones, which in turn are modulators of inflammation;
- Fatty acids can undergo oxidation processes and the compounds obtained can act on inflammatory cells by binding to specific receptors;
- Fatty acids can be incorporated into cell membranes where they keep the fluidity of the membranes intact; membrane phospholipids are also substrates for diacylglycerol, and fatty acids can act as transcription factors or precursors for the biosynthesis of lipid mediators.

## 4. Fatty Acid: Preclinical Studies

Several animal models were used to study the effects of EPA. As reported in a study conducted on a mouse model of hyperlipidemia, represented by ApoE- and LDL-receptor-deficient mice, the administration of EPA reduced the development of atherosclerotic lesions and increased the cell content of omega-3 PUFAs, without changing the total cholesterol or HDL content [25]. The analysis carried out on the atherosclerotic plaques of mice administered with EPA showed a stable morphology associated with a lower deposition of lipids and a reduced accumulation of macrophages accompanied by an increase in smooth muscle cells and collagen content. An anti-inflammatory effect of EPA was also highlighted due to the inhibition of the expression of adhesion molecules and monocyte chemoattractant protein 1 (MCP-1) and inhibition of the production of metalloproteinases by macrophages. The atherosclerotic plaques of mice treated with EPA had a stable morphology, including less lipid deposition, decreased accumulation of macrophages, increased smooth muscle cells, and greater collagen content. In addition, EPA had an anti-inflammatory effect on endothelial cells, inhibiting the expression of adhesion molecules and MCP-1 and by inhibiting production of matrix metalloproteinase by macrophages.

EPA is converted to 18R-hydroxyheicosapentaenoic acid (18R-HEPE) by COX-2-expressing vascular endothelial cells, which is acetylated in the presence of aspirin during inflammation. Endothelial cells expressing COX-2 convert EPA into 18R-HEPE, which is subsequently released by endothelial cells and converted via neutrophil-derived 5-LOX through a common epoxy intermediate in Resolvin E1 (RvE1) and Resolvin E2 (RvE2).

Data from a study in which they were used have recently been acquired. ApoE 3 Leiden transgenic mice transgenic (known as mice able to develop hyperlipidemia and susceptible to diet-induced atherosclerosis) were used to study RvE1 obtained by the EPA on the development of arteriosclerotic lesion. The animals were given a hypercholesterolemic diet for 9 weeks after which they received the administration of RvE1 (low or high dose) for 16 weeks. A group treated with atorvastatin and one with low-dose RvE1 were also added to the study atorvastatin. Compared to the control group, the group treated with RvE1 (low or high dose) and with atorvastatin reduced the arteriosclerotic lesion from 35% to 27%; the combination of RvE1 and atorvastatin reduced the atherosclerotic lesion area by 51% [26]. Data collected from another study conducted on LDL-receptor-deficient mice showed that EPA significantly reduces the size of atherosclerotic plaques as well as total cholesterol levels [27]. Levels of proinflammatory cytokines and chemokines (TNF alpha; interferon gamma) were lower than in the control group. Another target on which EPA acts is represented by dendritic cells whose phenotype is reorganized after administration of EPA; the number of T cells is reduced in the spleen and lymph nodes [28].

## 5. Fatty Acids: Clinical Studies

The importance of the use of omega-3 fatty acids in general, and of EPA in particular, is also linked to the numerous guidelines on their use; for the National Lipid Association (NLA) omega-3 fatty acids represent the first option for the treatment of patients with high triglyceride (TG) levels (≥500 mg/dL) and as an add-on option to statin therapy for those with high TG levels (200–499 mg/dL) [29]. For the Japan Atherosclerosis Society (JAS) the addition of EPA to a statin therapy represents an added value for the treatment of high-risk patients with LDL-C levels ≥140 mg/dL [30]. Numerous clinical studies have reported data on the importance assumed by omega-3 PUFAs on the prevention of ASCVD; in particular, on the international scene, four studies stand out: Gruppo Italiano per Io Studio della Sopravvivenza nell’Infarto Miocardico (GISSI)-Prevention study [3], the Japan Eicosapentaenoic Acid Lipid Intervention (JELIS trial) [31], the GISSI-Heart Failure study [32], and the Reduction of Cardiovascular Events with Icosapent Ethyl-Intervention Trial (REDUCE-IT) [33] (see Table 2). Moreover, other studies were also formulated in which a low dose of omega-3 PUFA was used (in a range of 840 mg/d of EPA + DHA) but which did not produce positive results. In particular, we can count the ORIGIN trial [34], the Risk and Prevention Study [35], the ALPHA OMEGA trial [36], A Study of Cardiovascular Events in Diabetes (ASCEND) trial [37], and the Vitamin D and Omega-3 Trial (VITAL) [38].

### EPA: Outcomes Studies

In the Outcomes to Assess Statin Residual Risk Reduction with Epanova in High CV Risk Patients with Hypertriglyceridemia (STRENGTH) study, the effects of a 4 g/day administration of EPA and DHA in a carboxylic acid formulation were analyzed [39]. A meta-analysis conducted on 20 studies that enrolled 68,680 patients showed that the administration of omega-3 PUFAs led to a reduction in the risk of cardiac death (RR 0.91, 95% CI: 0.85–0.98) in secondary prevention [40]. Data obtained from this survey led the American Heart Association (AHA) of 2017 to express a scientific opinion in which it is reported that the low-dose supplementation of omega-3 PUFA was conceivable to prevent the secondary manifestations of coronary diseases in patients with overt coronary heart disease. [41] A recent meta-analysis formulated by expanding the data with those obtained from three recent large-scale RCTs of omega-3 PUFAs (REDUCE-IT, ASCEND, and VITAL) clearly highlighted the beneficial properties of omega-3 PUFAs in limiting the risk of developing cardiovascular events. These reductions appear to be associated with the supplemental omega-3 PUFA dose [42]. In the analysis carried out by the REDUCE-IT study, the effects obtained from the use of the ethyl ester EPA (icosapent ethyl 4 g/day) on the development of high-risk cardiovascular events in patients on drug treatment with statins were examined [33]. The clinical condition of the patients was subsequently assessed with 4.9-year follow-up, which demonstrated a positive effect of using EPA [33]. Data obtained from REDUCE-IT led the National Lipid Association to recommend the administration of icosapent ethyl for patients aged ≥45 years with clinical ASCVD, or aged ≥50 years with diabetes mellitus requiring drugs plus ≥1 factor of additional risk, with fasting triglycerides 135 to 499 mg/dL on high-intensity or maximally tolerated statin therapy to reduce the risk of ASCVD [43]. The FDA has approved the administration of icosapent ethyl to both treat patients with triglyceride levels ≥500 mg/dL, and to reduce the risk of ASCVD in people with diabetes mellitus and two or more additional risk factors for cardiovascular disease in drug therapy with statins [43]. The difference between the positive effect of icosapent ethyl reported in the REDUCE-IT [33] study and the lack of an effect of the mixed carboxylic acids EPA and DHA in the STRENGTH [39] study is still questionable. The studies cited used omega-3 PUFAs at high doses but in different formulations, so comparisons should be made with caution. The studies were designed differently in at least three aspects. First, the omega-3 PUFAs were administered as an ethyl ester formula in the REDUCE-IT study and as non-esterified fatty acids, rapidly ionized transforming into molecules with detergent properties (soaps), in the STRENGTH study. Second, unlike the corn oil used in the STRENGTH study, the placebo mineral oil used in the REDUCE-IT study may have influenced expectations. Third, the DHA component of omega-3 PUFAs may be ineffective or even harmful, although there are currently no studies on the ASCVD outcomes of DHA monotherapy. The significant therapeutic efficacy of EPA in combination with statin on ASCVD was not found in other triglyceride-lowering agents, including fenofibrate and niacin, which failed to reduce cardiovascular events as compared to statin treatment alone [44,45]. With the withdrawal of recommendations by the FDA on the combination of statins with fibrates or niacin in the prevention or treatment of ASCVD, icosapent ethyl remains a viable non-LDL target therapy for patients with increased ASCVD risk and hyper-triglyceridemia. Pemafibrate to Reduce Cardiovascular Outcomes by Reducing Triglycerides in Patients with Diabetes (PROMINENT; NCT03071692), an ongoing trial of pemafibrate (a selective PPAR *α* modulator that significantly lowers triglyceride) in patients with type 2 diabetes mellitus, mild-to-moderate hypertriglyceridemia, and low HDL-cholesterol might further shed light on the mechanism of triglyceride-lowering agents on ASCVD [46].

## 6. Role of EPA on Athero-Inflammatory-Thrombotic Processes

**Figure 2:**
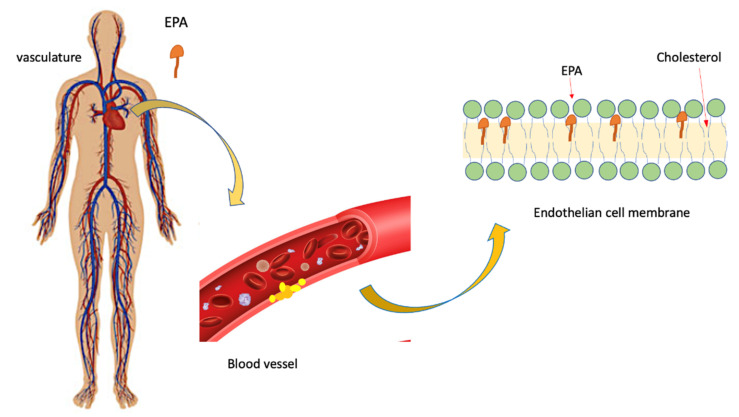
Possible effect of EPA on the endothelial membrane: inhibition of the propagation of free radicals. According to this model, it is hypothesized that EPA is able to intercalate between membrane phospholipids, in the central region, inhibiting the propagation of free radicals and thus preserving a more homogeneous distribution of cholesterol.

The scientific literature demonstrates how EPA plays a beneficial function in the regulation of endothelial tone. The endothelial cells, in fact, release nitric oxide (NO), which has the ability to modulate the vasomotor tone in response to acetylcholine and other vasoactive agonists [47]. Under physiological conditions, there is, in the body, an endothelium-dependent vasodilation due to the release of NO. If one has endothelial dysfunction, then NO release is reduced or absent. In this case, we are witnessing the appearance of toxic effects due to reactive oxygen costs, including peroxynitrite (ONOO_1) [47,48]. EPA is able to significantly reduce the formation of reactive oxygen species, as well as the expression of adhesion molecules, the release of pro-inflammatory cytokines, and the apoptotic cascade, as demonstrated by the Din in vitro studies conducted on HUVEC cells [49]. EPA was also found to be able to reduce or inhibit lipid peroxidation in membrane vesicles with even high cholesterol levels. The highlighted effect is also easy due to the presence of statins [44]. It is known that glucose contributes to the development of lipid peroxidation, the direct consequence of which is the appearance of high cholesterol levels. In this process, EPA also plays a key role, since it inhibits the formation of lipids [50]. The antioxidant effect of EPA seems to be due to its ability to intercalate in the lipid bilayer of the membrane while preserving its structural component (Figure 2) [50].

### EPA and Atherosclerotic Plaque

Recent studies show how EPA is able to reduce the neo vasculogenic process in human endothelial cells by acting on the modulation of the expression of the c-lit protein on the PI3-K/Akt/eNOS pathway, thus preventing ischemic injury [51]. Two studies in particular demonstrate these EPA actions; in the ANCHOR study, the administration of EPA at a dose of 4 g/day for 12 weeks reduced the oxidation of LDL by 13.3% compared to the placebo group in patients treated with statins and high triglycerides (from 200 to <500 mg/dL). In the MARINE study, there was a reduction of 6.6% (*P* = 0.055) in patients with higher levels of triglycerides (500 to <2000 mg/dL) [52]. In patients with type 2 diabetes mellitus and on drug therapy with statins, the addition of EPA at a dose of 1.8 g/day for 6 months) improved the endothelial function, as well as the conditions of CHD patients [53]. Similarly, in hyperlipidemic patients, the administration of EPA at a dose of 1.8 g/day for 3 months restored endothelium-dependent vasodilation at a superimposable level compared to that found in the group of normolipidemic patients [54]. Co-administration of EPA at a dose of 1.8 g/day for 48 weeks) with statins compared to statin monotherapy significantly inhibited the progression of arterial stiffness as observed in the analysis of the b-index stiffness parameter of the carotid artery in CHD patients (*p* = 0.02) [55]. EPA at a dose of 4 g/day for 12 weeks was shown to be effective in significantly reducing RLP-C by 25.8% in statin-treated patients who exhibited high levels of triglycerides (*p* = 0.0001) and 29.8%, respectively, in patients with even higher triglyceride levels (*p* = 0.0041) than in the placebo group [56]. Analyzing the ANCHOR study even more selectively, it was found that EPA significantly reduces RLP-C by 25.0% (*p* <0.0001) and VLDL triglycerides by 28.9% (*p* < 0.01) compared to the placebo group in the subgroup of patients with type 2 diabetes mellitus and on drug treatment with statins and elevated triglycerides [57]. In another study, in patients with type 2 diabetes mellitus and metabolic syndrome, EPA administered at a dose of 1.8 g/day for 3 months significantly reduced the volume of dense LDL particles (*p* < 0.01) and RLP triglycerides (*p* < 0.05) compared to baseline [58]. Numerous studies have shown that EPA is able to significantly reduce the volume of lipid plaque. By adding EPA at a dose of 1.8 g/day to background drug therapy with high-intensity statins, it significantly reduces the volume of lipid plaque and that of fibrotic plaque after 6 months (both *p* < 0.05) as measured by ultrasound intravascular [59]. Similarly, EPA at a dose of 1.8 g/day in co-administration with pitavastatin significantly reduced coronary plaque volume after 8 months of treatment compared to pitavastatin alone (−24% vs. −2%, *p* < 0.01) in patients with impaired glucose tolerance and angina pectoris [60]. In patients treated with EPA for 1 year, the tomography reported an important reduction in volume of soft plaque, contrary to what was seen in patients undergoing pharmacological treatment with ezetimibe [61]. The aforementioned studies have shown that the EPA, in general, exerts protective effects on the development of arteriosclerosis processes; in particular, it is clear in its involvement in the protection of endothelial function, in the reduction of oxidative stress levels, and in the maintenance of low inflammatory levels, which see the release of cytokines, platelet aggregation, and thrombus formation. Furthermore, EPA is also able to act on the reduction of atherogenic dyslipidemia levels with numerous benefits given by the ability to intercalate in the lipid bilayer of the plasma membrane. Of considerable pharmacological interest is the data that demonstrate how the beneficial effects of EPA are still maintained if administered together with statins. A clinical trial, randomized and controlled, called REDUCE-IT (NCT01492361), is now available, developed to evaluate the administration of EPA in a highly purified formulation [62]. The drug currently under study is called icosapent ethyl and represents a high purity formulation containing the ethyl ester of EPA. The recommended therapeutic dose in the trial is 4 g/day to be taken as two 1 g capsules twice daily with food. Therapeutic results were also achieved in the MARINE and ANCHOR studies. In particular, in the MARINE study, conducted in patients with elevated triglyceride levels, treatment with icosapent ethyl at a dose of 4 g/day for 12 weeks significantly reduced their levels by 33.1% (*p* < 0.0001), VLDL triglycerides by 25.8% (*p* = 0.0023), VLDL cholesterol (VLDL-C) by 28.6% (*p* = 0.0002), non-high density lipoprotein cholesterol (not HDL-C) by 17.7% (*p* < 0.0001), and Apo B by 8.5% (*p* = 0.0019) compared to the placebo values [63]. In the ANCHOR study conducted in patients on drug treatment with statins and elevated triglycerides, treatment with icosapent ethyl at a dose of 4 g/day for 12 weeks significantly reduced triglyceride levels by 21.5% (*p* < 0.0001), LDL-C by 6.2% (*p* = 0.0067), VLDL triglycerides by 26.5% (*p* < 0.0001), C-VLDL by 24.4% (*p* < 0.0001), C-non-HDL by 13.6% (*p* < 0.0001), and Apo B by 9.3% (*p* < 0.0001) compared to placebo [63]. On the basis of the data obtained from the analysis of the different clinical trials, a new pharmacoeconomic model was prepared that highlighted how the combination of EPA and statin for the secondary prevention of cardiovascular diseases is now associated with important economic savings and utility therapeutics when compared with single statin treatment [64].

## 7. Conclusions

Numerous studies have now shown that the inflammatory process is the basis of various diseases; in this context, it is now recognized that the role of fatty acids is to control inflammation by changing their composition in cell membranes, for example making it more fluid or altering its gene expression. The key cells of the inflammatory process are rich in arachidonic acid n-6 fatty acid, but their content can vary through oral administration of EPA and DHA. The increase in the membrane content of EPA and DHA causes a change in the production pattern of eicosanoids and probably also of resolvins. Given the involvement that n-3 marine PUFAs have in modulating inflammatory responses, it is understood how these can be decisive in inflammatory process and resolution. The clinical data obtained from the anti-inflammatory evaluations obtained thanks to the role of EPA have raised the awareness that an increase in the diet could bring a clinical benefit. There have been numerous studies that have provided encouraging data in patients with rheumatoid arthritis [65], though fewer in patients suffering from inflammatory bowel diseases [2]. With regard to these diseases, the data between adults and children are conflicting; in the latter, there are greater therapeutic successes [66]. A very interesting study was conducted in pregnant women where the administration of EPA determined beneficial effects on both the maternal and fetal immune systems [67,68], so as to reduce the risk of onset of allergic diseases during early childhood [69]. These first evidences opened the field to study the effects of EPA on the immune system [70]. In the plethora of inflammatory diseases, an exception is represented by cardiovascular disease, of which, to date, studies on EPA have been most concentrated [71,72], whose effects are particularly evident in the stabilization of atherosclerotic plaques [73]. A univocal and clear dose of EPA to be used has not yet been identified, although it is clear that the therapeutic effects are strictly dose-dependent [23]. Studies conducted on volunteers have highlighted that an intake of >2 g EPA + DHA/day is required to modulate inflammatory processes. Studies in patients with rheumatoid arthritis used 1.5 to 7 g EPA + DHA/day (average about 3.5 g/day) and were long-lasting (3 to 12 months), with effects which appear after several months [74]. One study compared two different doses [75], showing that the greatest benefits were obtained with the higher dose. Reading data clearly demonstrate that fatty acids modulate inflammatory phenomena through various motive mechanisms related to changes in the fatty acid composition of cell membranes. Inflammatory cells are rich in n-6 arachidonic acid, but the content of arachidonic acid and n-3 fatty acids EPA and DHA may vary after oral administration of EPA and DHA. The EPA in itself can be a source of eicosanoids with characteristics different from those deriving from arachidonic acid. In fact, both EPA and DHA are sources of recently discovered resolvins with anti-inflammatory properties. The altered fatty acid composition of inflammatory cells also alters the synthesis of peptide mediators of inflammation such as adhesion molecules or cytokines.

Summarizing, data related to clinical investigation reported the following:

- EPA lowers both triglyceride and cholesterol levels [7,8];
- Unlike DHA, EPA does not cause the increase of low-density lipoprotein cholesterol (LDL-C) levels [63,76];
- EPA protects against oxidative damage and improves endothelium formation [77];
- EPA inhibits monocyte movement into early lesions and subsequent conversion to macrophages and foam cells [78];
- EPA supports anti-oxidant and anti-inflammatory functions of high- density lipoprotein (HDL) [79];
- EPA promotes HDL-mediated cholesterol efflux from macrophages [79];
- EPA reduces atherosclerotic plaque formation, progression, and vulnerability to rupture [80];
- EPA decreases platelet-mediated thrombus formation [81];
- EPA reduces blood pressure, likely attributable to improvement of endothelial function [82]; importantly, many of these effects have been observed with EPA alone or are additive to those of statins.

## Figures and Tables

**Table 1:** PUFAs n-6 (or omega-3) family.

Sources of Dietary n-3 PUFA	Sources of Dietary n-3 PUFA	ALA (α-Linolenic Acid)	EPA (Eicosapentaenoic Acid)	DHA (Docosahexaenoic Acid)	Ref.
Fish oil	Menhaden (oil) Salmon (oil) Herring (oil) Sardine (oil)	- - - -	13.18 13.3 6.28 10.15	8.56 18.23 4.21 10.66	[10,11]
Fish raw	Salmon (raw) Sardine (raw) Cod (dried) Trout (raw) Herring (raw)	0.09 - - 0.1 0.19	0.89 0.51 0.02 0.15 1.09	1.19 1.16 0.62 0.5 1.01	[11]
Beef	New Zealand, liver (raw) New Zealand, kidney (cooked)	0.05 0.08	0.11 0.15	0.04 0.03	[10]
Oils	Soybean (oil) Wheat germ (oil) Sunflower (oil) Flaxseed (oil) Safflower (oil) Corn (oil) Canola (oil)	7.3 5.3 0.33 53.37 0.1 0.6 9.15	- - - - - - -	- - - - - - -	[10,11]
Seed and nuts	Chia (dried/ground) Hazelnut (dried/ground) Almond (dried/ground) Hemp seed (hulled) Brazil nuts (dried) Walnut (dried/ground)	17.83 0.11 0.3 8.68 0.02 6.64	- - - - - -	- - - - - -	[10,11]

**Table 2:** Clinical studies showed data on the relevance of omega-3 PUFAs on the prevention of ASCVD.

Clinical Trial	Patient Characteristics	Dose PUFA	Outcomes	Ref
(GISSI)-Prevention study	Men and women (15%) after myocardial infarction	850 mg EPA/DHA	The group treated with omega-3 PUFAs were shown to have a 20% reduction in major CV events, a 30% reduction of CV death, and a 45% reduction in SCD	[3]
JELIS trial	Hypercholesterolemic men and women (69%), with and without CHD, already receiving statin therapy	1800 mg EPA	Treatment was associated with a 19% reduction in major CV events	[29]
GISSI-Heart Failure study	Men and women (22%) with congestive heart failure	850 mg EPA/DHA	Treatment was associated with a 6% reduction in CV death or hospitalization	[30]
REDUCE-IT	Middle-aged, history of CVD or DM; TG 135–499 mg/dL; LDL-C 40–100 mg/dL with statin	4000 mg EPA	Treatment was associated with a reduction risk of ischemic events	[31]

## Data Availability

Not applicable.
